# Probing Deep Lung Regions using a New 6-electrode Tetrapolar Impedance Method

**DOI:** 10.2478/joeb-2022-0016

**Published:** 2023-01-08

**Authors:** Mahjabin Mobarak, Muhammad Abdul Kadir, K Siddique-e Rabbani

**Affiliations:** 1 Bangladesh University of Professionals, Dhaka, Bangladesh; 2 Department of Biomedical Physics and Technology, University of Dhaka, Dhaka, Bangladesh

**Keywords:** Deep lung bio-impedance, EIT, TPIM, 6-electrode TPIM, depth sensitivity

## Abstract

Probing deep regions of the lung using electrical impedance is very important considering the need for a low cost and simple technique, particularly for the low and medium income countries. Because of complexity and cost, Electrical Impedance Tomography is not suitable for this envisaged application. The simple Tetrapolar Impedance Measurement (TPIM) technique employing four electrodes is the age old technique for bioelectrical measurements. However, it has its limitations in respect of organ localisation and depth sensitivity using skin surface electrodes. Recently, a new 6-electrode TPIM with two current electrodes but two pairs of appropriately connected potential electrodes positioned on the front and back of the thorax, proposed by one of the authors, came with a promise. However, this work gave a qualitative proposal based on concepts of physics and lacked a quantitative evaluation. In order to evaluate the method quantitatively, the present work employed finite element method based COMSOL Multiphysics software and carried out simulation studies using this new 6-electrode TPIM and compared the results with those from 4-electrode TPIM, with electrodes applied either on the front or at the back of the thorax for the latter. Initially, it carried out a sensitivity distribution study using a simple rectangular volume conductor which showed that the 6-electrode TPIM gives better depth sensitivity throughout the lung region. Next it used a near life like thorax model developed by another of the authors earlier. Using this model, extensive studies were carried out to quantify the overall sensitivity over a target lung region, the contribution of the target lung to the total measured impedance, and several other parameters. Through these studies, the 6-electrode TPIM was established on a stronger footing for probing deep regions of the lungs.

## Introduction

The electrical impedance measured across the thorax at any specific measurement frequency changes significantly with the breathing cycle due to filling up of spaces within the lung by low conductivity air to varying degrees. Therefore, measurement of electrical impedance of lungs has a potential application in pulmonary medicine, particularly for the low and medium income countries (LMIC), for its technological simplicity resulting in low cost devices that are easy to apply and maintain.

The basic method for measuring bioelectrical impedance is the age old Tetrapolar Impedance Measurement (TPIM) technique, which uses four electrodes and can eliminate electrode contact impedance. This technique was introduced by William Thomson, popularly known as Lord Kelvin in 1861, to measure low resistances where the lead wire resistance and the contact resistance created problems [1]. Later, this technique was applied by Nyboer for bio-electrical measurements where the contact impedance is a major problem [2]. Since then it has been the standard measurement method in the measurement of biological tissues including the human body. Different derived techniques based on TPIM, such as Electrical Impedance Tomography (EIT) (originally named as Applied Potential Tomography, APT) [3] and Focused Impedance Method (FIM) [4,5] have evolved over time to provide better localisation of the measurement regions. All these methods were applied to study lung parameters during the breathing cycle by many researchers [6, 7, 8, 9] showing reasonable outcomes. EIT gives an image of the impedance distribution but is a rather complex technique which would be potentially expensive when commercially produced. On the other hand, TPIM and FIM are simple techniques which would result in potentially low cost devices and would be more appropriate for the LMICs. Recently, another simple technique, a 6-electrode TPIM was proposed by one of the authors [9], which was particularly aimed at getting improved sensitivity in the deeper regions of a volume conductor using surface electrodes employing the same electrical measurement setup of the simple TPIM.

**Fig.1:**
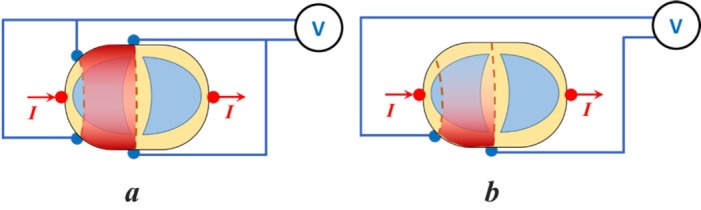
Electrode connections for transfer impedance measurement using the 6-electrode TPIM technique (left, 1a) and that for a 4 electrode TPIM (right, 1b) used for comparison. The sensitive regions are indicated in dark red, shades representing qualitative magnitudes (not to scale). Reproduced from [9] with permission.

The new 6-electrode TPIM is described here with the help of figure 1, reproduced from [9], which is particularly targeted to one lung. In this method, two current electrodes are fixed at the two sides (lateral points) of the thorax while two pairs of potential measuring electrodes are affixed on the front and back symmetrically over the region of interest (figure 1a), in the same horizontal plane. The corresponding potential measuring electrodes on the front and back are connected together. This was expected to give better sensitivity in the deep regions compared to a similar configuration of conventional 4-electrode TPIM having only one pair of potential electrodes on the front or at the back (figure 1b). Qualitative visualisations of the sensitivities of the two configurations are represented in these figures through shades of dark red. The idea is similar to that of a Helmholtz coil for producing a uniform magnetic field between two coils [10].

The above proposal for a 6-electrode TPIM was made based on concepts of physics, from qualitative visualisations based on concepts of equipotentials and sensitivity [11]. Subsequently, Roy et al. [12] studied this new 6-electrode TPIM on a cylindrical phantom, both through numerical simulation using a software package named COMSOL Multiphysics [13], which utilises the Finite Element Method (FEM), and through experimental measurements, and found improvement in the deep region sensitivity compared to that for the conventional 4-electrode TPIM configuration (figure 1b). However, this work was targeted to any volume conductor in general, not necessarily to any particular organ.

The present work particularly focused on the lungs which required changes in the shape and the design of the volume conductor.

## Materials and methods

**Fig.2:**
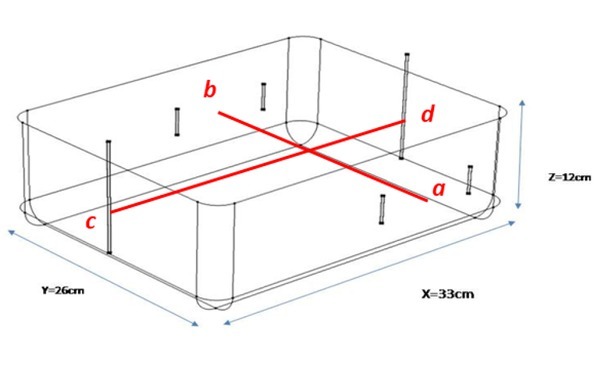
3D schematics of the simulated 6-electrode TPIM configuration in a rectangular volume conductor.

For an initial understanding of the point sensitivity pattern, a simple rectangular volume conductor with rounded edges (phantom) was simulated in COMSOL with electrodes for a 6-electrode TPIM targeting one side of the volume, over the region of a lung, as shown in figure 2. The dimensions of the volume were 33 cm × 26 cm × 12 cm corresponding to assumed x, y and z directions respectively as shown. Here the electrodes were long electrodes, current electrodes covering the full height while the potential electrodes were 3 cm long placed at vertical mid-level. All the electrodes had a width of 3 mm. The separation between the potential measuring electrodes, both in the front and the back, was 15 cm. For simulation of a 6-electrode TPIM and for a 4 electrode TPIM, electrical connections as shown in figure 1 were used. The volume conductor was supposed to be filled with a fluid with uniform a conductivity of 0.081 S/m.

Since the current drive and potential measuring electrodes are not the same in a TPIM, the measured impedance, given by *Z=V/I*, is usually called a ‘transfer impedance’ [14] to distinguish it from the simple bipolar impedance measurement based on Ohm’s Law.

The point sensitivity values were calculated using the following equation [11] which was based on lead fields introduced by Geselowitz [15]:


(1) $$S=\frac{J_{1}\cdot J_{2}}{I^{2}}$$

where $J_{1}\text{ and }J_{2}$are the current density vectors at a point within the volume conductor due to injection of the same current *I* through the exciting (current drive) and sensing (potential measuring) electrode pairs, respectively. This analysis draws its justification from the well-known reciprocity theorem [16] which essentially says that if the current and the potential electrode pairs are interchanged in any system, the measured values of transfer impedance remain unchanged.

The transfer impedance Z of the volume conductor is then given by,


(2) $$Z=\int_{V}\rho \;S\;\;dV$$

where *ρ* is the point impedivity and the integral is carried out over the full volume V. (Impedivity is the specific impedance, an equivalent to resistivity, which is the specific resistance.)

In order to visualise the point sensitivity distribution of the uniform volume conductor (phantom), colour contours in specific 2D planes were displayed while variations of numerical values were plotted along two lines *ab* and *cd* as indicated in figure 2, which lie centrally in the respective geometries.

**Fig.3:**
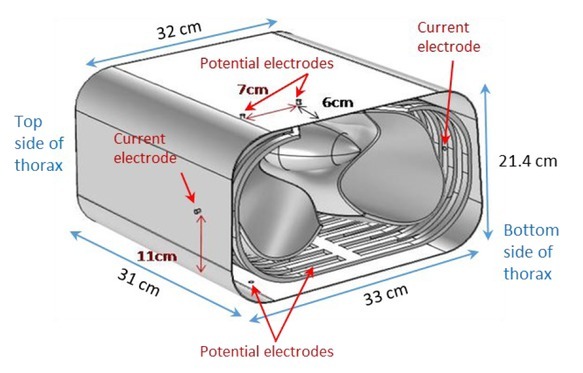
Near to real life 3D model of the thorax. The positioning of electrodes for the measurements undertaken are also shown. Reproduced from [17] with permission.

**Fig.4:**
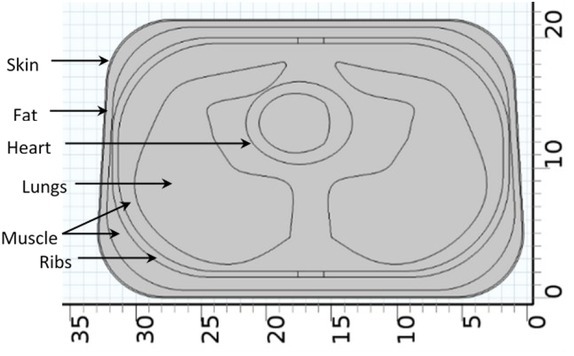
Some details of a cross section of the modelled thorax. Reproduced from [17] with permission.

Next, the efficacy of the 6-electrode TPIM technique in targeting deep regions of one lung was simulated using a near to real life 3D model of the thorax, as developed by one of the authors [17] and shown in figure 3. The dimensions of the thorax were estimated from typical CT images of an adult human male of height 170 cm. The overall depth and height of the modelled thorax were 21.4 cm and 31 cm respectively. This assumes a person standing. The width was 33 cm on the back and 32 cm on the front. The model incorporated skin, transcutaneous fat, ribs, muscle, heart filled with blood and lungs as indicated in figure 4. To build the overall model and to give it a realistic shape, various geometry operations of COMSOL Multiphysics were performed. The appropriate impedances for the different tissue types incorporated in the model were taken from a publication of the Italian Institute for Applied Physics [18].

The simulations were performed at full inspiration and full expiration of the lungs at a measuring frequency of 10 kHz. The conductivity and permittivity of the lung tissue at this frequency were obtained from the above publication which were 0.2429 S/m and 34044 respectively at full expiration and 0.093172 S/m and 17174 respectively at full inspiration.

Normally, the lung volume changes significantly with the breathing cycle, but for simplicity, in this modelled thorax, it was assumed constant. Similarly, the volumes of other organs around the lungs were assumed constant. Both the left and right lungs modelled were similar in shape and size and the volume of each lung was 2030 cm^3^. The details are available in the original work [17].

For simplicity, the thorax model was considered as piecewise homogeneous and isotropic. This means that any domain within the model was assumed to be homogeneous and isotropic, but the electrical properties of different domains are different. Boundary conditions were set so that the current density on the outer boundary of the thorax is zero whereas the boundaries between internal domains satisfy continuity.

Circular stainless steel electrodes of 0.3 cm diameter were taken to touch the outer skin surface for all the electrodes (Figure 3). The electrode plane was taken at a height of 6 cm from the bottom of the thorax (in the vertical direction of the thorax, for a standing posture) for the present study. The current electrodes were fixed on the sides at about the midpoint of the depth of the thorax. The potential electrodes, on both the front and back surfaces, were positioned as shown in figure 3, with a 7 cm separation for the initial study. The potential electrode nearest to the side of the body was at a distance of 6.5 cm from the front edge of the thorax. The outcome of this study showed the presence of negative sensitivity regions at the corners of the horizontal cross section of the lungs (showed in the results section, figure 8). With a view to reduce this negative sensitivity region, a second study was taken up for which the potential electrode nearest to the side of the body was moved laterally to a new position at a distance of 3.5 cm from the front edge of the thorax. The inner electrode was kept fixed in position. This created an electrode separation of 10 cm (measured on the surface of the thorax) between the potential electrodes on both the front and back.

**Fig.5:**
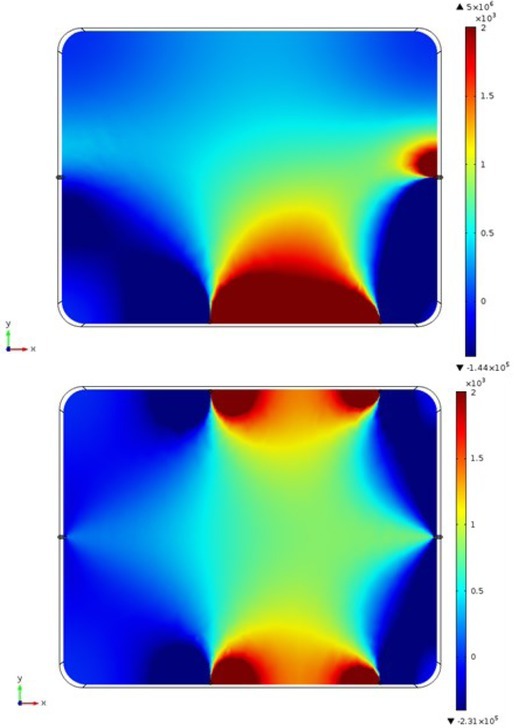
Sensitivity distribution along XY (horizontal) plane at for Z=6 cm for 4-electrode TPIM (top) and 6-electrode TPIM (bottom) with potential electrode separation of 15cm. Sensitivity range truncated beyond ^−^400 and +2000.

**Fig.6:**
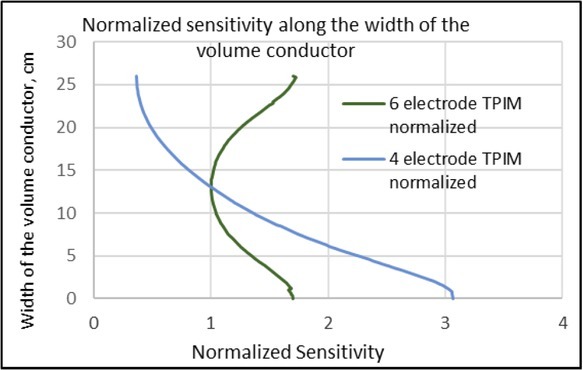
Normalised sensitivity values along the depth *(ab)* for 6-electrode TPIM and 4-electrode TPIM for the simple volume conductor model shown in Figure 2. That for the former has much better uniformity at the deep regions compared to that for the latter.

**Fig.7:**
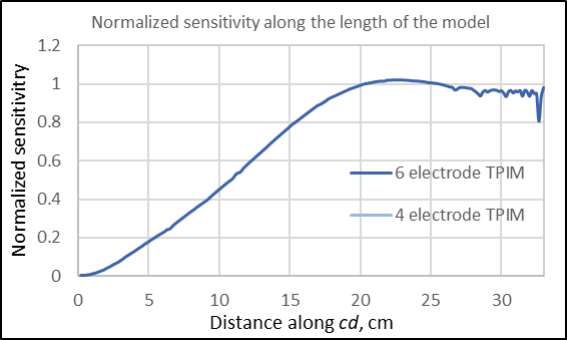
Normalised sensitivity values along the width *(cd)* for 6-electrode TPIM and 4-electrode TPIM for the simple volume conductor model shown in Figure 2. Both have exactly the same sensitivity, with the highest at the cross point of *ab* and *cd*.

**Fig.8:**
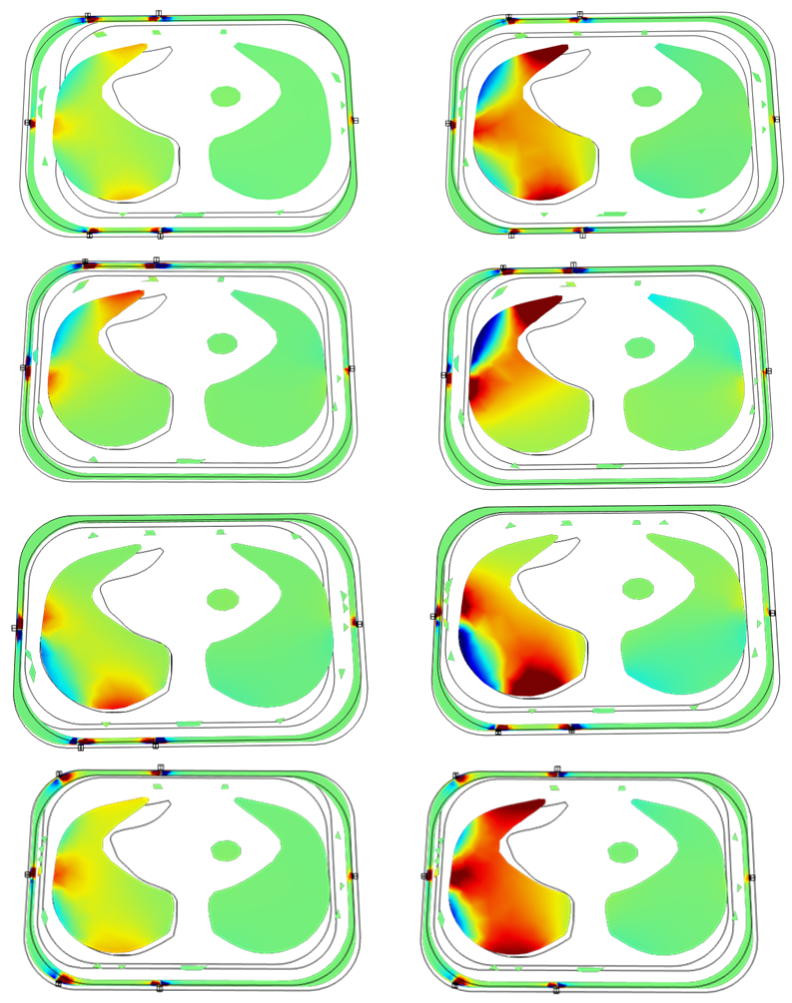
Sensitivity distribution over the lungs for 6-electrode TPIM (6-TPIM) and 4 electrode TPIM (4-TPIM) for inspired lung (left column) and expired lung (right column) in the cross sectional plane of the electrodes, as obtained for the life like thorax model with different Electrode Separations (ES). Shades of brown indicate higher sensitivity, darker for higher values, while shades of blue indicate regions having negative sensitivity. *1st row*: 6-TPIM, ES=7 cm, *2^nd^ row*: 4-TPIM, potential electrodes on front, ES=7 cm, *3^rd^ row*: 4-TPIM, potential electrodes on back, ES=7 cm, *4^th^ row*: 6-TPIM, ES=10 cm.

Equation 2 gives the total transfer impedance for the whole volume of the thorax in which the respective impedivity of each point is considered according to the assumed thorax model. For convenience, ‘transfer impedance’ is shortened to simply ‘impedance’ henceforth. Since the total impedance will change during respiration, the total impedance change (TIC), expressed in the form of a percentage, was defined for this study as,


(3) $$TIC=\frac{Total\;\;impedance(inspired)-Total\;\;impedance(expired)}{Total\;\;impedance(expired)}$$

Obviously, one would like to have a configuration giving a maximum value of the above parameter.

Since the lung is the target organ in this present study, the contribution of the target lung to the total impedance, CLTI, is an interesting and useful parameter. Any measurement targeting the lung should try to maximise CLTI. Depending on the target lung region, either both or anyone, CLTI may be adapted to consider the appropriate lung volume.

Thus if *Z_T_* is the total impedance as given by equation 2, integrated over the whole thorax volume, and *Z_L_* is the impedance of the target lung region, obtained through an integration of equation 2 only for the respective target lung volume, then CLTI is given by,


(4) $$CLTI=\frac{z_{L}}{z_{T}}$$

Here, *Z_T_* includes *Z_L_*. CLTI was expressed in percentage.

In this work, two versions of CLTI were used. In one, named CLTI(1), the integration for *Z_L_* was performed over only the target lung volume, around which the electrodes are placed. For the second, named CLTI(2), the integration was performed over both the lung volumes even though the electrodes were positioned to target one lung only. A comparison of CLTI(1) with CLTI(2) will give an idea of the degree of localization achieved through the electrode configurations as described above.

The overestimations in CLTI of a target lung due to the other lung (OCLTI) was obtained as,


(5) $$OCLTI=\frac{CLTI(2)}{CLTI(1)}$$

OCLTI was expressed in percentage and would indicate the success of the electrode placement in focusing the target lung. A low value of OCLTI is desired which would indicate a small contribution from the other lung outside the potential electrode configuration.

### Ethical approval

The conducted research is not related to either human or animal use and so no ethical approval was necessary.

## Results and Observations

### Sensitivity in the rectangular volume conductor

Figure 5 shows the sensitivity distributions over a horizontal cross section of the volume conductor described in Figure 2 for a front-sided 4 electrode TPIM (top) and for the recently proposed 6-electrode TPIM (bottom), for an electrode separation of 15 cm between the potential electrode pair on each side. It is to be noted that the maximum and minimum sensitivity values differ between these two studies. Therefore, in order to give a visual qualitative comparison, the sensitivity ranges for display in both the figures were truncated below -400 and above +2000.

Figure 5 shows the improvement achieved through the new 6-electrode TPIM which gives higher sensitivity near both the front and back while the 4 Electrode version gives high sensitivity only on the front (or only on the back if the potential electrodes on the back were used). The main point is that the sensitivity in the deep region has less variation for the 6-electrode TPIM (better uniformity), compared to that for the one sided 4 electrode TPIM.

Next, normalised numerical sensitivity values along the depth (along *ab* in Figure 2), are shown in Figure 6. The values were normalised to the corresponding values at the midpoint of the line *ab*. It may be noted that while the sensitivity for the one sided 4 electrode TPIM varies through a factor of 8.3, the 6-electrode TPIM has far less variation, a factor of only 1.7, a big improvement.

It may further be noted that the absolute value of the sensitivity for the 4 electrode TPIM at the front surface (not shown here) was almost double that of the corresponding value for the 6-electrode TPIM. This happened since the current delivered in both the models was the same and the current through the potential electrodes as given in equation 1 for the sensitivity equation was divided equally between the two pairs at the front and the back for the 6-electrode TPIM, while it was solely driven through a single pair for the 4-electrode TPIM. However, it is the uniformity of the sensitivity one is after, not the absolute value, and definitely 6-electrode TPIM gives a much better performance than the conventional 4-electrode TPIM, as originally conceived. It may also be envisaged that with a greater electrode separation, the uniformity over the depth may be better.

Figure 7 shows the normalised sensitivity variation for the two configurations of electrodes along the width (line *cd* in Figure 2), normalised at the cross-point of *ab* and *cd*. For this, the two curves are exactly identical and hence these overlap totally. The highest value occurs at the above mentioned cross point, which is expected.

### Studies on the real life like thorax model

Figure 8 shows the sensitivity pattern in the two lung regions only for both the 6-electrode TPIM and the 4-electrode TPIM over the cross sectional plane of the electrodes as shown in Figure 3 (6 cm from the base of the thorax). The sensitivity distributions are shown for both inspired and expired conditions of lungs and with potential electrodes on the front and on the back separately for 4-electrode TPIM as indicated in the figure caption. The 1^st^ three rows had the electrode separation between the potential electrodes on each side as 7 cm while for the 4^th^ row it was 10 cm. Shades of brown indicate higher sensitivity, more for darker shades, while shades of blue indicate negative sensitivity in a similar way. It is to be noted that in negative sensitivity regions, a change towards higher impedivity will show as a reduction in measured impedance and vice versa for a change towards lower impedivity.

From the diagrams in figure 8 the following observations may be of interest:

- For each measurement configuration, the sensitivity was higher in the target lung around which the potential electrodes are placed, compared to that in the other lung.
- For each measurement configuration, the overall sensitivity distribution was less during inspiration compared to that during expiration. This is expected since during inspiration the impedivity of the lungs is higher than that at expiration, so less current lines will go through the lung regions in the former case compared to the latter.
- 6-electrode TPIM gives reasonable sensitivity distributed almost over the whole of the target lung while 4-electrode TPIM gives high sensitivity only in the region near the potential electrodes.
- For 4-electrode TPIM, that from the back covers more of the lung region than that from the front. This is expected because of the anatomical shape and size of the lung.

For 7 cm electrode separation areas of negative sensitivity can be seen in the target lung region in figure 8, indicated by the blue regions between the current and potential electrodes, which is a normal consequence of TPIM. If the negative sensitivity region covers a large area of the lungs, the net contribution of the lungs to the measured impedance will be reduced, contributing to an error. In order to study if the area of the negative sensitivity could be reduced by small changes, the potential electrodes near the edge of the thorax were shifted by 3 cm towards the edge, both on the front and back, keeping the other electrode fixed. This gave a separation of 10 cm (measured on the thorax surface) between the potential electrodes both on the front and the back of the thorax and the resulting sensitivity distribution for the 6-electrode TPIM only is shown in the 4^th^ row of figure 8. Compared with the pattern in the 1^st^ row, it is clear that the areas of the negative sensitivity regions have indeed decreased.

However, one needs to remember that the above patterns shown in Figure 8 correspond to that in the electrode plane only. In the total measured impedance, the contribution of the lungs will come from the whole lung volume. However, the planar distribution shown above is indicative and may be used to visualise the effect of the whole volume.

The total impedance at inspiration and expiration for the two different separations of potential electrodes and the corresponding contributions of the lungs to the total impedance for the target lung and for both the lungs together are shown in Tables 1 and 2, respectively. The overestimations in CLTI of a target lung due to the other lung, given by OCLTI, are also shown.

**Table 1:** Simulation results for potential electrode separation of 7 cm

	6-TPIM			4-TPIM, Front			4-TPIM, back		
	Inspired	Expired	% change*	Inspired	Expired	% change*	Inspired	Expired	% change*
Total Imp, Ω	10.6	7.8	34.7	10.4	8.0	30.4	10.6	7.7	37.9
% CLTI(1)	24.2	35.6	-32.1	20.5	31.2	-34.1	28.3	40.4	-29.8
% CLTI(2)	25.0	38.0	-34.1	22.2	35.3	-37.1	28.2	41.2	-31.5
% OCLTI	3.6	6.8		8.2	13.4		-0.3	2.1	

* with respect to expired condition

CLTI(1): Contribution of target Lung to total impedance

CLTI(2): Contribution of both Lungs to total impedance

OCLTI: Overestimation in CLTI of the target lung due to the other lung

**Table 2:** Simulation results for potential electrode separation of 10 cm

	6-TPIM			4-TPIM, Front			4-TPIM, back		
	Inspired	Expired	% change*	Inspired	Expired	% change*	Inspired	Expired	% change*
Total Imp, Ω	13.9	10.6	31.4	13.6	10.3	32.2	14.0	9.9	42.1
% CLTI(1)	26.3	36.4	-27.6	21.9	33.4	-34.4	29.8	42.8	-30.5
% CLTI(2)	27.2	38.0	-28.6	23.5	36.9	-36.3	29.8	43.5	-31.4
% OCLTI	3.2	4.6		7.4	10.6		0.2	1.6	

* with respect to expired condition

For a potential electrode separation of 7 cm (Table 1), it may be observed that percentage changes in total impedance are above 30% for all the three electrode configurations, being the highest for 4-TPIM at back. CLTI from the target lung at inspiration varies between 20.5% and 28.3% while that at expiration varies between 31.2% and 40.4%, variations having the same trend for the different electrode configurations. The overestimation parameter OCLTI is also lowest for 4-TPIM at back and highest for 4-TPIM at the front, with that for 6-electrode TPIM in-between.

It may also be observed that CLTI for an expired lung is greater than that for an inspired lung. This is because more current lines converge into the lung region in the expired condition when the impedance of the lung regions is considerably lower than that in the inspired condition, as expected.

Table 2 shows similar parameters as in Table 1 but for a potential electrode separation of 10 cm as described before. The changes in total impedance between inspiration and expiration have slight but mixed changes. However, of special interest were the changes in %CLTI(1) and %OCLTI achieved by increasing the separation from 7 cm to 10 cm, for both inspiration and expiration, which are presented in Table 3 as ratio of values for 10 cm Electrode Separation to those for 7 cm. It may be observed that %CLTI(1) values increased slightly for all the measurements shown, indicating a small improvement. However, the values of the overestimation parameter %OCLTI were reduced considerably, showing a better performance with 10 cm electrode separation.

**Table 3:** Ratio of values for 10 cm Electrode Separation to those for 7 cm ES

	6-TPIM	4-TPIM Front	4-TPIM back
% CLTI(1) Inspiration	1.09	1.07	1.05
% CLTI(1) Expiration	1.02	1.07	1.06
%OCLTI Inspiration	0.91	0.90	
%OCLTI Expiration	0.68	0.79	0.75

## Discussion

This work presents a quantitative evaluation of a new technique of 6-electrode TPIM introduced by one of the authors [9], and this work particularly targeted the lungs, using finite element simulation, employing the COMSOL software package.

Firstly, the sensitivity distribution within a rectangular volume for a particular separation between the potential electrodes was studied and the results shown in figure 5 clearly shows the improvement produced. The sensitivity of the one sided 4-electrode TPIM remains limited to one side of the volume while for the 6-electrode TPIM the sensitivity extends through the whole depth. The normalised sensitivity distributions along the depth of the volume at the midpoint of the potential electrode configuration were shown in figure 6. This also shows clearly that the variation of sensitivity along the depth for 6-electrode TPIM (by a factor of about 1.7) is much reduced compared to that (about 8.3) for 4-electrode TPIM. The main point to note is that the 4-electrode TPIM is only good for organs at shallow depths near the potential electrodes, while for organs like the lungs, extending almost through the depth, 6-electrode TPIM is a better choice.

Figure 7 shows the normalised sensitivity along the width of the rectangular volume, where both the 6-electrode TPIM and the 4-electrode TPIM have exactly the same pattern, which is expected. The sensitivity has a very low value on the left edge (since the potential electrodes in this case were placed on the right hand side of the volume as shown in figure 2) and it goes through a maximum at the midpoint of the potential electrode configuration, which is also expected.

**Fig.9:**
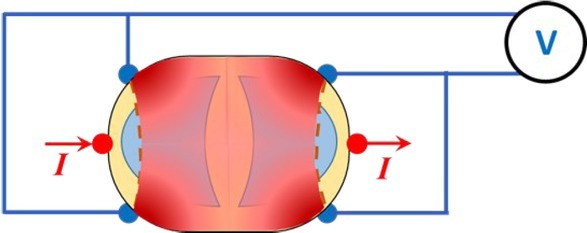
6-electrode TPIM covering both lung regions. Reproduced from [9] with permission.

It may be anticipated that the uniformity with depth would improve through increasing the separation between the potential electrodes on each side, however, depending on the geometry of the target organ one may have to make a trade off. Looking at Figure 1, the electrodes were placed to target one lung only. However, if one intends to cover both the lungs, the two potential electrodes may be positioned near the two edges of the thorax when the uniformity with depth would be better. This was also indicated by Rabbani [9] and reproduced in Figure 9. Of course, in this case the heart with low impedivity blood will take a good share of the current distribution. However, for any measurement on lungs, one usually looks for changes during respiration and the contribution of the heart may be separated since the rates of the two changes are normally different and they are not synchronised.

The life like thorax model developed by one of the authors [17] adds a new dimension to this work. It has modelled the main tissues and organs within the thorax including the heart filled with blood. However, the volume of lung is assumed to be constant during respiration, for simplicity, which is a limitation. This approximation will have a bearing on the outcome of the present work as well. In future, if the lung volume is changed appropriately in the model, the simulation quality will improve. Again, the heart volume is also assumed to be constant, which needs appropriate changes in the future. However, since the beating of the heart and respiration have different rates and are not synchronous, as mentioned above, the assumed fixed heart will not affect the outcome of the present work significantly.

The improvement in depth sensitivity using the new 6-electrode TPIM over a conventional 4-electrode TPIM is adequately demonstrated through the sensitivity distributions obtained using the above mentioned life like thorax model as presented in Figure 8. The 1^st^ row and the last row show how the whole depth of the lung contributes to the measurements. On the other hand, the 4-electrode TPIM either on the front or on the back only covers the respective regions down to a certain depth. However, it is apparent that 4-electrode TPIM placed at the back covers a greater volume of the target lung because of the anatomy and geometry of the lung itself.

For the 1^st^ to 3^rd^ row of Figure 8, the separation between the potential electrodes on each side was 7 cm, as shown in Figure 3. This shows presence of small regions of negative sensitivity within the target lung, on the left side of the figure, which would underestimate the contribution from the lung in the impedance measurement. Negative sensitivity regions occur in between the current and voltage electrodes in a TPIM measurement. Intuitively, shifting the electrodes near the edges further towards the edge may reduce the negative sensitivity region. Based on this intuition, these electrodes were shifted by 3 cm over the surface of the thorax as a trial, increasing the separation between the potential electrodes to 10 cm. The 4^th^ row in Figure 8 gives a visual indication, only for the 6-electrode TPIM, of the improvement thus obtained. Table-3 shows this improvement numerically, comparing the corresponding values presented in Tables 1 and 2. For 6-electrode TPIM, the improvement in %CLTI(1) was small at expiration, a mere 2%, but at inspiration, the improvement was higher at 9%. The figures for the 4-electrode TPIMs were between 5% and 7%. However, the improvement in overestimation error due to the presence of the other lung is substantial, particularly for expiration. This shows that the intuitive logic behind shifting the electrodes near the edges worked. However, further studies are required to get an optimum separation.

A point worth noting from Figure 8 is that at inspiration, the overall sensitivity in the target lung region is much less than that at expiration. This is due to higher impedivity of lungs at inspiration when the current lines divert away to outer tissues in the thorax, while at expiration, more current lines converge into the lungs increasing its contribution. An assessment of the magnitudes involved is presented by the numerical values presented in Tables 1 and 2. From Table 2, for 10 cm separation between the potential electrodes, CLTI(1) of the target lung at inspiration stood at 26.3% for 6-electrode TPIM, 21.9% for 4-electrode TPIM at front, and 29.8% for 4-electrode TPIM at back. The corresponding figures at expiration were, 36.4%, 33.4% and 42.8% respectively. The reduction of the corresponding values at inspiration with respect to that at expiration stood at 27.6%, 34.4% and 20.5% respectively. These are significant changes and it is important that one considers this effect when using electrical impedance for diagnosis of the lungs.

Another point studied was the overestimations in CLTI of a target lung due to the other lung, given by OCLTI. The idea is that when one lung is targeted through a particular electrode configuration, it is desirable that no contribution should come from the other lung that is not targeted. In any lung disorder the two lungs may have different physiological conditions when the contribution from the other lung may lead to erroneous inferences. This parameter is shown numerically in Tables 1 and 2 for 7 cm and 10 cm separation of potential electrodes respectively. For 6-electrode TPIM, this overestimation stood at 3.6% and 6.8% respectively at inspiration and expiration for 7 cm electrode separation, and the corresponding values were 3.2% and 4.6% respectively for 10 cm electrode separation. The values are similar for the other TPIM configurations. These figures are very low, indicating that there is a very low contribution from the other lung in both these configurations, which is a success of this arrangement.

Again, to assess the improvement obtained using potential electrode separation of 10 cm over that for 7 cm, Table 3 shows the ratios of the values for %CLTI(1) and %OCLTI both for inspiration and expiration. For %CLTI(1) the ratios varied between by 2% to 9%, which indicate only small improvements. However, there was a considerable reduction in %OCLTI ratio, particularly for expiration, which were between 0.68 and 0.79 for the different TPIM configurations indicating a marked improvement with 10 cm separation over 7 cm separation. At inspiration, the corresponding ratios were not that low, about 0.9 for both 6-electrode TPIM, and 4-electrode TPIM (frontal), indicating a small improvement. However, for the 4-electrode TPIM at back, the overestimation at inspiration changed signs and so the ratio was not shown. For this case, it can be seen from Tables 1 and 2 that for 7 cm separation, the other lung contributed negatively by 0.3% while for 10 cm separation there was a positive contribution of 0.2%. Both these values are very small and negligible. However, the negative value suggests the presence of a very small negative sensitivity region in the other lung.

Looking at the OCLTI values in Tables 1 and 2, 4-electrode TPIM applied at the front gives high overestimation, but that the back gives very low values, even lower than that for 6-electrode TPIM, indicating low contributions from the other lung. From this viewpoint 4-electrode TPIM applied at the back may give the best performance, however, going back to the sensitivities shown in figure 8, 4-electrode TPIM does not cover the whole depth of the lungs, which is covered by the 6-electrode TPIM configuration. Therefore, if only the back side of the lungs is of interest, 4-electrode TPIM may be chosen, but if the whole depth of the lungs is of interest, which is normally the case in any diagnosis, 6-electrode TPIM would be the better choice.

The present work establishes the recently proposed 6-electrode TPIM on a stringer footing for application in real life.
